# Four new synonyms and a new combination in *Parnassia* (Celastraceae)

**DOI:** 10.3897/phytokeys.77.11513

**Published:** 2017-03-15

**Authors:** Yumin Shu, Zhixiang Zhang

**Affiliations:** 1 Laboratory of Systematic Evolution and Biogeography of Woody Plants, College of Nature Conservation, Beijing Forestry University, Beijing 100083, Chinas; 2 Museum of Beijing Forestry University, Beijing 100083, China

**Keywords:** Taxonomy, lectotypification, *Parnassia*, morphology

## Abstract

*Parnassia yunnanensis* had been previously described based on mixed specimens containing materials partially belonging to *Parnassia cacuminum*, which makes the application of *Parnassia yunnanensis* ambiguous. Therefore, we lectotypified *Parnassia yunnanensis* and meanwhile synonymized Parnassia lanceolata var. oblongipetala under it. Parnassia yunnanensis var. longistipitata was found more similar to *Parnassia cacuminum* rather than *Parnassia yunnanensis*, thus a new combination, Parnassia cacuminum var. longistipitata **comb. nov.** was proposed. Furthermore, other three names (*Parnassia vevusta*, *Parnassia degeensis* and *Parnassia kangdingensis*) were reduced to synonyms of *Parnassia cacuminum* too.

## Introduction

*Parnassia* L. is a genus containing approximately 70 species (Ku and Hultgård 2001) which are predominantly distributed in arctic and temperate zones of the northern hemisphere and are mostly diverse in China and the Himalayas (Simmons 2004).

**Figure 1.**
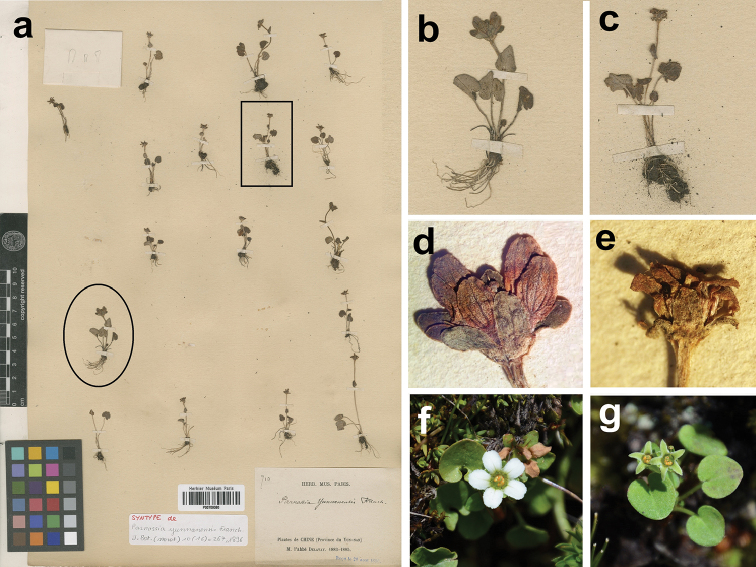
A mixed type specimen of *Parnassia yunnanensis*. The sheet of *Delavay 710* with barcode P 00709380 (**a**), the individual indicated with a circle and magnified should be determined as *Parnassia cacuminum* (**b, d**), an individual represents the rest, indicated with a square and magnified (**c, e**), *Parnassia cacuminum* (**f**) and *Parnassia yunnanensis* (**g**) in the wild.

*Parnassia yunnanensis* Franch. (1896: 266) was described from Heqing, Yunnan province in China in 1896 which based on two collections (*Delavay 710* and *Delavay s.n.*). Four duplicates of *Delavay 710* were successfully traced from P and K (herbaria acronyms following Thiers 2016). However, one of these duplicates deposited in P (barcode number P00709380, Figure 1a) is a mixed specimen: an individual indicated by a circle bears obovate petals (magnified as Figure 1b, d), while the rest possess lanceolate petals (Figure 1c, e, magnified from the individual indicated by a square). Obviously, they are different taxa, the individual with obovate petals should be *Parnassia cacuminum* Hand.-Mazz. (1931: 433). Franchet used ‘oblonga’ to describe the shape of petals in the protologue of *Parnassia yunnanensis*, that made this name ambiguous, some new names were published to be closely related to *Parnassia yunnanensis* but were actually close to or conspecific with itself or *Parnassia cacuminum*.

Pan described *Parnassia longipetaloides* J.T. Pan (1985: 222) based on *Jinshajing Exped. 6105* in KUN collected from Eryuan, Yunnan in 1963 and this name was placed in synonymy of *Parnassia yunnanensis* in *Flora Yunnanica* (Chuang 2006). According to *T.T. Yu 7429A* collected from Muli, Sichuan province in 1937, *Parnassia lanceolata* T.C. Ku (1987: 34) was described by Ku and soon after, she proposed a new variety, Parnassia lanceolata var. oblongipetala T.C. Ku (1991: 82) based on an isotype of *Parnassia longipetaloides*, *Jinshajing Exped. 6105* stored in PE. Wu et al. (2008) reduced *Parnassia lanceolata* to a synonym of *Parnassia yunnanensis*, but they did not mention var. oblongipetala.

Parnassia yunnanensis var. longistipitata Z.P. Jien (1963: 255) was proposed in 1963. This variety has white petals which are significantly different from *Parnassia yunnanensis* but closer to *Parnassia cacuminum*. *Parnassia venusta* Z.P. Jien (1963: 257), *Parnassia degeensis* T.C. Ku (1987: 30) and *Parnassia kangdingensis* T.C. Ku (1987: 35) are three taxa that are morphologically close to *Parnassia cacuminum*, but the authors compared them to some other taxa in the protologues when published them. Further studies in collections and field expeditions have been carried out to synonymise them in this paper.

## Methods

This paper is based on the critical review of the protologues and examination of specimens in herbaria BJFC, CDBI, K, KUN, P, PE, HNWP, SM, SZ and online on Jstor Global Plants (https://plants.jstor.org/ accessed on 12 December 2016). The measurements provided herein were mostly taken from dried herbarium specimens, and certain features such as colours were supplemented with the information from field observation. By applying Art 9.1 and 9.2 of the ICN (McNeill et al. 2012) strictly, the lectotype was selected. Specimens examined were listed alphabetically.

## Results and discussion

Since the syntypes of *Parnassia yunnanensis* belong to more than one taxon, a lectotype needs to be designated for it. Wu et al. (2008) cited the type of *Parnassia yunnanensis* in their taxonomic work as “*China. Yunnan, Hokin, Delavay 710 (holotype K!); Sichuan, near Tatchienlu, Pratt 542 (syntype, BM!)*”. As Franchet did not mention *Pratt 542* in the protologue when he described this new name, *Pratt 542* should not be regarded as a syntype of *Parnassia yunnanensis*. In addition, they did not use the phrase “designated here” or the term “lectotype” in the statement. According to Art. 7.10 and 9.23 of the ICN, their typification of *Parnassia yunnanensis* was not effective. Franchet worked at P (Stafleu and Cowan 1976), so we select a well-presented specimen with barcode P00709378 that contains most individuals as the lectotype.

**Figure 2.**
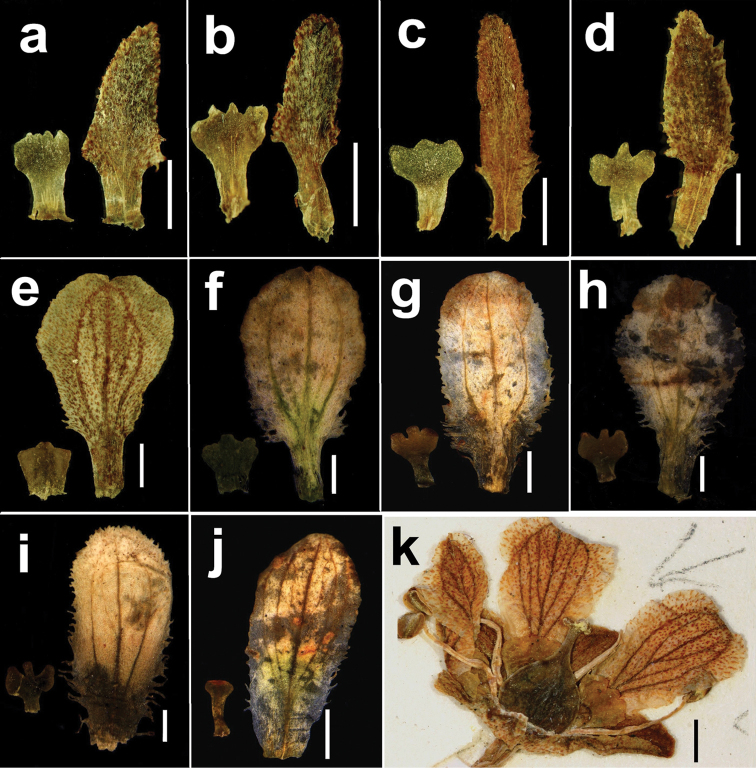
Morphology of the petals and staminodes in *Parnassia*. Parnassia lanceolata var. oblongipetala (**a, b** *Jinshajinag Exped. 6105*, the holotype), *Parnassia yunnanensis* (**c, d** *T.T. Yu 7429*), *Parnassia cacuminum* (**e** *T.T. Yu 12254* **f** *Y.M. Shu et al. sz286*), *Parnassia kangdingensis* (**g, h** *Y.M. Shu et al. sw365*), *Parnassia venusta* (**i** *Y.M. Shu et al. sw365*), Parnassia cacuminum var. longistipitata (**j** *Y.M. Shu et al. sw459*), *Parnassia degeensis* (**k** *Y.W. Tsui 4997*, the holotype). Scale bar = 1 mm.

Ku stated in the protologue of Parnassia lanceolata var. oblongipetala that it differs from the typical variety in having oblong petals, obtuse at the apex, and *Parnassia lanceolata* differs from *Parnassia longipetaloides* in having 3-lobed staminodes while the latter have obscure 4–6 dentate ones. By taxonomic revision of this variety, we determined that the type specimen of this name was indistinguishable from that of *Parnassia yunnanensis* in terms of taxonomically important morphological characters (shapes of petals and staminodes, Figures 1g, 2a–d) and their general distribution ranges (both type specimens were collected from Mt. Maer between Hoqing and Eryuan). Thus we treated this variety as a new synonym of *Parnassia yunnanensis*.

*Parnassia venusta* was described based on *T.T. Yu 22666* collected from Gongshan, Yunnan by Jien. As concluded from the protologue, he thought that this species differs from *Parnassia cacuminum* in two characters: leaves reniform, petals lanceolate-obovate, slightly fimbriated at the base (Figure 2i) while *Parnassia cacuminum* has cordata leaves, petals late obovate, erose or slightly fimbriated at the base (Figures 1f, 2e, f). The difference in the shape of the leaves was observed and we found it do not held good; reniform to cordate leaves always occur within a population even within a single individual. *Parnassia cacuminum* has obovate petals, but it is variant, 4–9 mm in length by 2–5 mm in width are observed in the sample specimens, and no dividing line can be found amongst lanceolate-obovate, obovate and late obovate. Hence, we treated *Parnassia venusta* as a new synonym of *Parnassia cacuminum*.

In 1987, Ku proposed two new species, *Parnassia degeensis* and *Parnassia kangdingensis* from Dege and Kangding respectively. *Parnassia degeensis* was described based on *Y.W. Tsui 4997*, which has only one sheet containing one individual with two flowers. Ku described the staminodes of this name as undulate or 5–7 dentates at the apex and compared it with *Parnassia farreri* W.E. Evans (1921: 174), which differed significantly in the shape of petals. We examined the holotype of *Parnassia degeensis* (Figure 2k); the petals were obovate with distinct veins, staminodes 3 lobed at the apex and that agreed well with *Parnassia cacuminum*. Ku stressed in the protologue of *Parnassia kangdingensis* that this new taxon was closely related to *Parnassia lanceolata*. However, from the description, it could be concluded that this name was quite different from *Parnassia lanceolata* (obovata white petal vs. lanceolata green petal) but close to *Parnassia cacuminum* by the numerical characters which Ku used to distinguish them, such as heights of individuals and length of petals. As we examined the type specimens and observed the populations in Kangding (Figure 2g, h), variations of height from 2–9 cm and length of petals from 3–6 mm were found within a population. Therefore, we propose to recognise *Parnassia degeensis* and *Parnassia kangdingensis* as synonyms of *Parnassia cacuminum*.

Not far from the location where *Parnassia kangdingensis* was described in Kangding, a new variety, Parnassia yunnanensis var. longistipitata was proposed. This variety has cordate leaves, elliptic to obovate white petals and flat, linear staminodes with entire or obscure 3 dentate apexes (Figure 2j). All these characters made it different from *Parnassia yunnanensis* but close to *Parnassia cacuminum*. The only difference between it and *Parnassia cacuminum* is the linear shaped staminodes. The field observation found that these linear shaped staminodes were easily distinguished from those of *Parnassia cacuminum* and they were stable within populations. Thus, it was proposed to recognise this taxon as a variety of *Parnassia cacuminum*.

### Taxonomic treatment

#### Parnassia yunnanensis

Taxon classificationPlantaeCelastralesCelastraceae

Franch., J. Bot. (Morot) 10(16): 266. 1896.

Parnassia longipetaloides J.T. Pan, Acta Phytotax. Sin. 23(3): 222. 1985. Type: China. Yunnan: Eryuan, Mt. Maer, elev. 3600 m, 25 Jul 1963, Jinshajinag Exped. 6105 (holotype: KUN [KUN0437666!]; isotype: KUN!).
Parnassia lanceolata T.C. Ku, Bull. Bot. Res. 7(1): 34. 1987. Type: China. Sichuan: Muli, elev. 3900 m, 28 Jul 1937, T.T. Yu 7429A (holotype: PE [PE01842934!]; isotypes: PE!, KUN!, E [image!]).
Parnassia lanceolata T.C. Ku var. oblongipetala T.C. Ku, Acta Phytotax. Sin. 29(1): 82. 1991. Type: China. Yunnan: Eryuan, Mt. Maer, elev. 3600 m, 25 Jul 1963, Jinshajinag Exped. 6105, **syn. nov.** (holotype: PE [PE01842922!]).

##### Type.

China.Yunnan: Heqing (Hokin), Kowa-la-po, in the shrub, 26 Aug 1884, Delavay 710 (Lectotype, designated here: P [P00709378!]; isolectotypes: P [P00709379!, P00709380! (exclude the individual with obovate petals)], K [K000739471!]).

##### Specimens examined.

**CHINA. Gansu**: Lintan, 26 Jul 2013, C. Shang I”-218 (BJFC); **Sichuan**: Jiulong, elev. 3800 m, 15 Jul 1979, T.C. Wei 20512 (CDBI); Jiulong, elev. 3800 m, 25 Aug 1980, Z.A. Liu 22993 (CDBI); Kangding, elev. 4010 m, 5 Aug 2015, Y.M. Shu et al. sw447 (BJFC); Kangding, elev. 3600 m, 6 Jul 1974, N.Z. Zhang 4801 (PE, CDBI); Kangding, elev. 3080 m, 25 Jul 1934, C.S. Liu 883 (PE, SZ); **Yunnan**: Dali, elev. 3800 m, 31 Jul 2014, Y.M. Shu et al. sz072 (BJFC); Dongchuan, 18 Jul 2009, Huang 1529 (KUN); Lijiang, elev. 4000 m, 17 Aug 2003, D. Wu et al. 3005 (KUN); Lijiang, elev. 4000 m, 26 Aug 2002, D. Wu et al. 2005 (KUN).

#### Parnassia cacuminum

Taxon classificationPlantaeCelastralesCelastraceae

Hand.-Mazz., Symb. Sin. 7(2): 433. 1931.

Parnassia venusta Z.P. Jien, Acta Phytotax. Sin. 8(3): 257. 1963. Type: China. Yunnan: Gonshan Hsien, Sawalunba, elev. 4000 m, 3 Sep 1938, T.T. Yu 22666, **syn. nov.** (holotype: PE [PE01842930!]; isotypes: PE!, KUN!).
Parnassia degeensis T.C. Ku, Bull. Bot. Res. 7(1): 30. 1987. Type: China. Sichuan: Dege, Haizikou, 24 Jul 1951, Y.W. Tsui 4997, **syn. nov.** (holotype: PE [PE01896065!]).
Parnassia kangdingensis T.C. Ku, Bull. Bot. Res. 7(1): 35. 1987. Type: China. Sichuan: Kangding, 28 Jul 1951, W.P. Fang et al. 10632, **syn. nov.** (holotype: PE [PE01842917!]; isotype: SZ!).

##### Type.

China. Sichuan: Muli, elev. 4450–4500 m, 30 Jul 1915, Handel-Mazzetti 7338 (holotype: WU [WU0046641 image!])

##### Specimens examined.

**CHINA. Qinghai**: Yushu, elev. 4250 m, 23 July 1964, Yushu Exped. 587 (HNWP, PE); **Sichuan**: Baoxing, elev. 4000 m, 1 Aug 2015, Y.M. Shu et al. sw365 et sw370 (BJFC); Baoxing, elev. 4048 m, 2 Jul 2010, C.S. Chang et al. SI0933 (PE); Daofu, elev. 3100 m, 17 Jul 1979, s.n. 1203 (SM); Dege, elev. 4000 m, 2 Jul 1979, s.n. 311 (SM); Dege, elev. 4100 m, 23 Jun 1974, Sichuan Zhibei Exped. 7121(CDBI); Dege, elev. 4300 m, 19 Jun 1974, Qingzang Exped. 63 (PE); Dege, elev. 4100 m, 23 Jun 1974, s.n. 7121 (PE); Jiulong, elev. 4110 m, 19 Jul 2012, L. He et al. PH20120719-03 (BJFC); Jiulong, 30 Jun 1974, Z.G. Liu 4730 (PE, CDBI); Jiulong, elev. 4100 m, 16 Jun 1984, W.L. Chen et al. 6345 (PE); Kangding, elev. 3730 m, 15 Jul 2012, L. He et al. PH20120715-06 (BJFC); Kangding, elev. 3700 m, 20 Jun 1984, W.L. Chen et al. 6541 (PE); Kangding, C.S. Liu 940 (PE); Kangding, 1 Aug 1963, West Sichuan Exped. 1414 (PE); Luhuo, elev. 3900 m, 5 Jul 1974, Q.H. Li et al. 6446 (PE, CDBI); Muli, 18 Aug 1937, T.T. Yu 7795 (KUN); Muli, Jun 1928, J.F. Rock 16564 (IBSC); Muli, elev. 4333 m, May 1932, J.F. Rock 23749 (PE); Muli, elev. 3700 m, 21 Jun 1937, T.T. Yu 6528 (PE, KUN); Muli, elev. 3400 m, 18 Aug 1937, T.T. Yu 7795 (PE); Rangtang, elev. 4300 m, 16 Jul 1979, s.n. 783 (SM); **Xizang**: Bomi, elev. 4300 m, 5 Sep 1982, B.S. Li et al. 00673 (PE); Chayu, elev. 3800 m, 8 Sep 1982, Qingzang Exped. 10118 (PE); Chayu, elev. 4100 m, 9 Sep 1982, Qingzang Exped. 10231 (PE, KUN); Chayu, elev. 3800 m, 19 Jul 2010, X.H. Jin et al. STET0551 (PE); Chayu, elev. 4100 m, 16 Jul 2010, X.H. Jin et al. STET0806 (PE); Chayu, elev. 3700 m, Aug 1935, T.W. Wang 65980 (PE); Cuona, elev. 4500 m, 19 Jul 1975, Z.Y. Wu et al. 75-1119 (KUN); Gongjue, elev. 4220 m, 15 Aug 2010, Kangzang Exped. 10-1966 (PE); Milin, elev. 4300 m, 28 Jul 1983, B.S. Li et al. 5960 (PE); Lasa, elev. 4952 m, 8 Jul 2012, L.M. Gao GLM-123788 (KUN); Linzhi, 23 Jul 2014, L. He PH20140723-10 (BJFC); **Yunnan**: Bijiang, elev. 4300 m, 12 Sep1964, S.K. Wu 8790 et 8810 (KUN); Deqin, elev. 4300 m, 19 Jul 2014, Y.M. Shu et al. sz 286 (BJFC); Deqin, elev. 4300 m, 31 Jul 2003, H. Wang et al. 3066 (KUN); Deqin, elev. 4300 m, 15 Jul 2004, J. Cai et al. 4209 (KUN); Deqin, elev. 4100 m, 10 Jul 1983, Hengduan Mt. Exped. 4576 (PE); Deqin, elev. 3200 m, Aug 1935, C.W. Wang 64943 et 64974 (PE); Deqin, elev. 3500 m, 6 Jul 1937, T.T. Yu 8758 (PE, KUN); Deqin, elev. 4000 m, 9 Sep 1938, T.T. Yu 22245 (PE, KUN); Deqin, elev. 3600 m, 4 Aug 1940, K.M. Feng 5921 (KUN); Deqin, 15 Aug 1976, J.S Yang 8573 (KUN); Deqin, elev. 3800 m, 1 Aug 1940, K.M. Feng 6209 (KUN); Eryuan, elev. 3700 m, 20 Jul 1963, Jinshajiang Exped. 63-6118 (KUN); Lijiang, elev. 4100 m, 14 Aug 2005, D. Wu et al. 5012 (KUN); Qiaojia, elev. 3800 m, 17 Jul 1973, B.X. Sun et al. 1032 (KUN); Weixi, elev. 3500 m, Jul 1935, C.W. Wang 64660 (PE); Weixi, elev. 3600 m, Aug 1935, C.W. Wang 68611 (PE); Xiangelila, elev. 4658 m, 3 Sep 2010, Kangzang Exped. 10-3237 (PE); Xiangelila, elev. 3600 m, 18 Jul 1937, T.T. Yu 12254 (PE, KUN); Xiangelila, 25 Jul 1939, K.M. Feng 1802 (PE); Xiangelila, elev. 4000 m, 6 Jul 2006, W.B. Yu et al. 5052 et 5053 (KUN); Xiangelila, elev. 3700 m, 12 Jul 1937, T.T. Yu 12085 (PE, KUN); Xiangelila, elev. 4000 m, 25 Jul 2003, D. Wu et al. 3028 (KUN); Yangbi, elev. 2600 m, 30 Jul 2003, D. Wu et al. 326 (KUN).

#### Parnassia cacuminum Hand.-Mazz. var. longistipitata

Taxon classificationPlantaeCelastralesCelastraceae

(Z.P. Jien.) Y.M.Shu & Z.X.Zhang comb. nov.

urn:lsid:ipni.org:names:77161383-1

##### Basionym.

Parnassia yunnanensis Franch. var. longistipitata Z.P. Jien, Acta Phytotax. Sin. 8(3): 255. 1963.

##### Type.

China. Sichuan: Kangding, Mt. Che To, 6 Aug 1934, C.S. Liu 1028 (holotype: PE [PE01842918!])

##### Specimens examined.

**CHINA. Sichuan**: Kangding, elev. ca. 4200 m, 6 Aug 2015, Y.M. Shu et al. sw459 et sw463 (BJFC); Kangding, elev. 4187 m, 12 Aug 2015, Y.M. Shu et al. sw505 (BJFC); Kangding, 7 Aug 2011, S.X. Yu 5008 (PE); Kangding, 10 Aug 2009, WPW 108 (KUN); Kangding, elev. 4000 m, 14 Jul 1981, Z.J. Zhao 114906 (SZ); Qianning, elev. 4300 m, 4 Jul 1974, Sichuan zhibei Exped. 5497 (PE, CDBI).

## Supplementary Material

XML Treatment for Parnassia yunnanensis

XML Treatment for Parnassia cacuminum

XML Treatment for Parnassia cacuminum Hand.-Mazz. var. longistipitata
